# InCoB2013 introduces Systems Biology as a major conference theme

**DOI:** 10.1186/1752-0509-7-S3-S1

**Published:** 2013-11-04

**Authors:** Christian Schönbach, Bairong Shen, Tin Wee Tan, Shoba Ranganathan

**Affiliations:** 1 Department of Biology, School of Science and Technology, Nazarbayev University, Astana 010000, Republic of Kazakhstan; 2 Department of Bioscience and Bioinformatics and Biomedical Informatics R&D Center (BMIRC), Kyushu Institute of Technology, Fukuoka 820-8502, Japan; 3 Center for Systems Biology, Soochow University, Suzhou 215006, PR China; 4 Department of Biochemistry, Yong Loo Lin School of Medicine, National University of Singapore, Singapore 117597, Republic of Singapore; 5 Department of Chemistry and Biomolecular Sciences and ARC Centre of Excellence in Bioinformatics, Macquarie University, Sydney NSW 2109, Australia

## Abstract

The Asia-Pacific Bioinformatics Network (APBioNet) held the first International Conference on Bioinformatics (InCoB) in Bangkok in 2002 to promote North-South networking. Commencing as a forum for Asia-Pacific researchers to interact with and learn from with scientists of developed countries, InCoB has become a major regional bioinformatics conference, with participants from the region as well as North America and Europe. Since 2006, InCoB has selected the best submissions for publication in *BMC Bioinformatics*. In response to the growth and maturation of data-driven approaches, InCoB added *BMC Genomics *in 2009 and with the introduction of this conference supplement, *BMC Systems Biology *to its journal choices for submitting authors. Co-hosting InCoB2013 with the second International Conference for Translational Bioinformatics (ICTBI) is in line with InCoB's support for the current trend in taking bioinformatics to the bedside, along with a systems approach to solving biological problems.

## Overview

Founded in 1998, Asia-Pacific Bioinformatics Network (APBioNet) is the oldest regional bioinformatics society in the Asia-Pacific and the first regional affiliate of International Society for Computational Biology (ISCB). APBioNet aims to promote and support bioinformatics within its region on educational, research and network infrastructure levels as a discipline firmly anchored in life sciences. In 2002, APBioNet supported the launch of the International Conference on Bioinformatics (InCoB) which evolved into its annual signature event [1]. This year InCoB was held in conjunction with the 2^nd ^International conference on Translational Biomedical Informatics (ICTBI) hosted by the Center for Systems Biology, Soochow University and Taicang Center for Translational Bioinformatics, China.

Renowned keynote speakers Wen-Hsiung Li (University of Chicago and Academia Sinica), Manyuan Long (University of Chicago) and Philip R.O. Payne (Ohio State University) presented and discussed topics ranging from intricacies of genome assembly methodologies to advances and obstacles in translational bioinformatics. Of special interest to the audience was Phillip Payne's 4i big data approach in conjunction with personalized medicine. The 4i include international systems-level data to information to knowledge management, scalable and high through-put data integration with improved human-computer interactions and constant innovation.

Of 108 submitted research papers, 45 (41%) were accepted after one to two rounds of peer-review by a 113 member strong Program Committee and 20 sub-reviewers (listed in Additional File 1) and subsequent revisions. The accepted manuscripts are published in *BMC Genomics *[2], *BMC Bioinformatics *[3], *BMC Systems Biology *and in the new open access journal PeerJ [4]. Both *BMC Systems Biology *and PeerJ were added this year to InCoB's choice of journals as a result of increasingly diverse topics of submitted papers in the fields of systems biology and biological image analysis. For the first time, oral paper presentations and posters were also offered the opportunity to submit their presentations under Common Creative License to F1000Posters [5]. Deposition with F1000Posters secures permanent availability of the authors' presentations and promotion of their research. The 10 articles published in this supplement are outlined below, arranged according to session topics.

### Literature mining and understanding disease

Two literature mining contributions introduced new tools in support of understanding pathway and disease relationships. PathNER [6] detects pathway names mentioned in the literature in context of a particular disease [7] whereas GenoMesh [8] predicts microbial gene relationships and genome-wide networks using similarity and dissimilarity functions [9]. The utility and impact of PathNER garnered the authors the InCoB Best Paper Award among manuscripts accepted for this supplement issue.

### ncRNAs and PPIs in regulation of cell differentiation

Four papers covered the control of cell differentiation and proliferation ranging from viewpoints of non-coding RNAs to temporal states of protein expression and interactions. Bagatov *et al*. [10] analysed the expression behaviour of long non-coding RNAs (lncRNAs) in retinoic acid induced differentiation of neuroblastoma cell line SH-SY5Y. Three major waves of coordinated lncRNAs and neighboring protein-coding genes expression were detected. Another paper utilizing genomic knowledge in combination with gene expression information focused on the survival prediction of glioblastoma multiforme (GBM) patients [11]. The results indicate that the use of inter-relationship information of miRNA expression and genomic features such as DNA methylation and copy number variation has the potential to elucidate tumorigenesis processes and improve therapeutic strategies for cancers other than GBM. Tang *et al*. [12] evaluated miRNA expression in prostate cancer using various statistical methods. Outlier robust T-statistics which showed the best performance provided support for two potentially new ESR1 and ESR2 activation pathways. Gong [13] targeted with his Symbolic Model Checking approach that incorporates dynamics of protein expression, protein-protein interactions and cell fate multi-gene targeted therapies of pancreatic cancer that require the understanding of interactions between pancreatic cancer cells and stellate cells. The P53-MDM2 and VEGF pathways along mutations in seven proteins were identified as critical in the uncontrolled proliferation of prostate cancer and stellate cells.

### Transcription regulation

Most computational efforts to characterize the promoter architecture in higher organisms have excluded plants. López *et al*. [14] developed a support vector machine-based method to predict promoter-specific regulator motifs of genes expressed in flower, seed, root or shoot of *A. thaliana*. The approach that incorporates motif positions, orientation within transcription factor binding sites and distance from translation start sites appears to be sufficiently generic to predict the broader, common promoter architectures among genes expressed in different structures of different metazoans. Chumnanpuen *et al*. [15] performed integrated analyses of transcription factors controlling lipid biosynthesis and the lipidome in yeast. Using nutrient limitation conditions, the authors were able to discern metabolic fluxes towards different lipid components.

### Drug-drug interactions

Two papers addressed drug-drug interactions from view point of functional drug groups and pharmacokinetics. In the drug development process the outcome of physiologically based pharmacokinetic (PBPK) simulations are used to determine the need of clinical drug-drug interactions studies. Lee *et al*. [16] focussed on networks among functional drug groups whereas Yoshida and co-workers [17] developed a PBPK model for the chemotherapeutic drug irinotecan. The model parameters were estimated with an improved Cluster Newton Method that allows for both parameter control and correlation.

### Towards molecular robotics

On the last conference day, Akihiko Konagaya (Tokyo Institute of Technology) introduced the development of molecular robots equipped with sensors and intelligence. The project is funded by a MEXT Grant-in-Aid for Scientific Research on Innovative Areas. The fundamental concept of the selfness of living systems and bottom-up self-organization is applied to robotics which traditionally follows top-down approaches [18]. Using a video, he demonstrated students' efforts to bring DNA molecular robots to the next level of complexity by introducing a compartment of a lipid bilayer and movement control. Over the next five years a molecular robotics consortium of four research teams [19] aims to develop robots with amoeba-like functions up to multi-cellular and hybrid robots. We encourage student teams from the Asia-Pacific as well as the rest of the world to follow this exciting new development and participate in future competitions in this area.

## Future directions of InCoB

Until recently APBioNet was organized in legal terms informally to provide maximum flexibility while keeping cost at minimum and providing membership for free. The resulting dynamics served APBioNet and its community well to grow and attain international competitiveness in bioinformatics. In 2012, APBioNet incorporated in Singapore as non-profit public limited liability company to sustain a high-quality regional support and provide a network to bioinformaticians that voids the gap between national societies with limited objectives and overarching goals of international societies. A modest annual membership fee structure is being developed to add new benefit for our members, which may also be collected through future InCoB registration fees. Value-added benefits will include various levels of discounted lifetime membership with PeerJ [20] a new open source publishing service allowing members to submit at least once a year for free, as of 2013. The addition of PeerJ to our traditional Supplement issues in BMC journals is expected to increase the number of registrations and oral presentations at future InCoB conferences. Unlike 'exploitative conferences' that publish any submission as long as the authors pay for them, APBioNet adheres to a revenue-neutral model that does not compromise the quality of peer-review.

In 2014 InCoB will be held for the first time in Australia. The three-day 13^th ^InCoB [21] will commence as the official Satellite of the 18^th ^International Union of Pure and Applied Biophysics (IUPAB 2014) congress [22] in Sydney, Brighton Beach on July 31, 2014.

## Competing interests

The authors were organizers and/or co-chairs InCoB2013. TWT is the Representative Director of Asia Pacific Bioinformatics Network, Ltd. All authors declare they have no other conflict of interest.

## Authors' contributions

CS and SR wrote this editorial. CS, BS, TWT and SR served as co-editors for the InCoB2013 supplement issues with SR as the lead editor. CS and SR managed the manuscript submission, peer-review and editorial decision processes as superchairs of the EasyChair Conference System. All authors have seen and approved this article.

## Supplementary Material

Additional file 1 **List of InCoB2013 Program Committee members and sub-reviewers**. Click here for file
