# Supplementary material for: InCoB2013 introduces Systems Biology as a major conference theme
Source: BMC Syst Biol. 2013 Nov 4;7(Suppl 3):S1. doi: 10.1186/1752-0509-7-S3-S1 (PMC3816296; doi:10.1186/1752-0509-7-S3-S1)
Supplement: Additional file 1 — List of InCoB2013 Program Committee members and sub-reviewers. [file 1752-0509-7-S3-S1-S1.PDF]

Additional File 1. List of InCoB2013 Program Committee members and sub-reviewers

Schönbach *et al*. BMC Systems Biology 2013, 7(Suppl 3): S1

We thank the members of the InCoB2013 Scientific Program Committee and twenty sub-reviewers for their constructive criticism and timely submission of review reports on manuscripts submitted to InCoB2013 supplement issues of BMC Systems Biology, BMC Genomics or BMC Bioinformatics.

<u>InCoB2013 Scientific Program Committee:</u>

- Shandar Ahmad (National Institute of Biomedical Innovation, Japan)
- Tatsuya Akutsu (Kyoto University, Japan)
- Shunsuke Aoki (Kyushu Institute of Technology, Japan)
- Nicola Armstrong (Garvan Institute of Medical Research, Australia)
- Vladimir Bajic (King Abdullah University of Science and Technology, Saudi Arabia)
- Christopher Baker (University of New Brunswick, Canada)
- Sergio Baranzini University of California at San Francisco, USA)
- Arsen Batagov (Bioinformatics Institute, A*STAR, Singapore)
- Alex Bateman (European Bioinformatics Institute, UK)
- Vladimir Brusic (Dana-Farber Cancer Institute, USA)
- Zhi-Wei Cao (Shanghai Center for Bioinformatics Information Technology, China)
- Filippo Castiglione (National Research Council of Italy, Italy)
- Jonathan Chan (King Mongkut's University of Technology Thonburi, Thailand)
- Ming Chen (Zhejiang University, China)
- Jiajia Chen (Suzhou University of Science & Technology, China)
- Wai-Ki Ching (The University of Hong Kong, SAR Hong Kong)
- Qinghua Cui (Peking University, China)
- Ning Deng (Zhejiang University, China)
- Frank Eisenhaber (Bioinformatics Institute, A*STAR, Singapore)
- Mahmoud Elhefnawi (National Research Centre, Egypt)
- Mohd Firdaus-Raih (Universiti Kebangsaan Malaysia)
- Andrew French University of Nottingham, UK)
- Ge Gao (Peking University, China)
- Pascale Gaudet (Swiss Institute of Bioinformatics, Switzerland)
- Humberto Gonzalez Diaz (IKERBASQUE Basque Foundation for Science, Spain)
- Marsia Gustiananda (Eijkman Institute for Molecular Biology, Indonesia)
- Tim Hancock (Kyoto University, Japan)
- Matthew He (Nova Southeastern University, USA)
- Yongqun He (University of Michigan, USA)
- Chia-Lang Hsu, National Taiwan University, Taiwan)
- Guang Hu (Soochow University, China)

- Jun Huan (The University of Kansas, USA)
- Kun Huang (The Ohio State University, USA)
- Chun-His Huang, University of Connecticut, USA)
- Ming-Jing Hwang (Academia Sinica, Taiwan)
- Chen Jiajia (Soochow University, China)
- Asif M. Khan (Perdana University, Malaysia)
- Javed Khan (Proteomics International, Australia)
- Tsung Fei Khang (University of Malaya, Malaysia)
- Daisuke Kiga (Tokyo Institute of Technology, Japan)
- Akira Kinjo (Osaka University, Japan)
- Akihiko Konagaya (Tokyo Institute of Technology, Japan)
- Shinji Kondo (National Institute of Polar Research, Japan)
- Anton Kratz (RIKEN Omics Science Center, Japan)
- Gaurav Kumar (Virgina Commonwealth University, USA)
- Igor Kurochkin (Bioinformatics Institute, A*STAR, Singapore)
- Chee Keong Kwoh (Nanyang Technological University, Singapore)
- Jinyan Li (University of Technology, Sydney, Australia)
- Xiaoli Li (Institute for Infocomm Research, A*STAR, Singapore)
- Guo-Zheng Li (Tongji University, China)
- Ao Li (University of Science and Technology of China, China)
- Guozheng Li, Wei Lin (Fudan University, China)
- Lei Liu (CAS Key Laboratory of System Biology, China)
- Xinghua Lu (University of Pittsburgh, USA)
- Yves A. Lussier (University of Chicago, USA)
- Hiroshi Mamitsuka (Kyoto University, Japan)
- Xizeng Mao (University of Georgia, USA)
- Hideo Matsuda (Osaka University, Japan)
- Bui Quang Minh (Max F. Perutz Laboratories, Austria)
- Lenny Moise (University of Rhode Island, USA)
- Santo Motta (University of Catania, Italy)
- Kenta Nakai (The University of Tokyo, Japan)
- Yasushi Okazaki (Saitama Medical University, Japan)
- Francesco Pappalardo (University of Catania, Italy)
- Ashwini Patil (The University of Tokyo, Japan)
- Nikolai Petrovsky (Flinders Medical Centre, Australia)
- Jiang Qian (Johns Hopkins School of Medicine, USA)
- Shoba Ranganathan (Macquarie University, Australia & National University of Singapore, Singapore)
- Timothy Ravasi (King Abdullah University of Science and Technology, Saudi Arabia)

- Yasubumi Sakakibara (Keio University, Japan)
- Meena Sakharkar, Tsukuba University)
- Daniele Santoni (National Research Council of Italy, Italy)
- Christian Schönbach (Kyushu Institute of Technology, Japan)
- Shahir Shamsir (University Teknologi Malaysia, Malaysia)
- Bairong Shen (Soochow University, China)
- Tetsuo Shibuya (The University of Tokyo, Japan)
- Narayanaswamy Srinivasan (Indian Institute of Science, India)
- Durai Sundar (Indian Institute of Technology Delhi, India)
- Y-H. Taguchi (Chuo University, Japan)
- Takako Takai-Igarashi (Tohoku Medical Megabank Organization, Japan)
- Yoichi Takenaka (Osaka University, Japan)
- Martti Tammi (University of Malaya, Malaysia)
- Tin Wee Tan (National University of Singapore, Singapore)
- Weidong Tian (Fudan University, China)
- Paolo Tieri (University of Bologna, Italy)
- Joo Chuan Tong (Institute of High Performance Computing, A*STAR, Singapore)
- Sissades Tongsima (National Center for Genetic Engineering and Biotechnology, Thailand)
- Ikuo Uchiyama (National Institute for Basic Biology, Japan)
- Chandra Verma (Bioinformatics Institute, A*STAR, Singapore)
- Mauno Vihinen (Lund University, Sweden)
- Jin Wang (Nanjing University, China)
- Yong Wan (Academy of Mathematics and Systems Science, China)
- Junbai Wang (Radium Hospital, Norway)
- Guohua Wang (Harbin Institute of Technology, China)
- Xiujie Wang (CAS, Institute for Genetics and Developmental Biology, China)
- Yufeng Wang (University of Texas at San Antonio, USA)
- Martin Wasser (Bioinformatics Institute, A*STAR, Singapore)
- Dongqing Wei (Shanghai Jiaotong University, China)
- Gonghong Wei (University of Oulu, Finland)
- Limsoon Wong (National University of Singapore, Singapore)
- Jingfa Xiao (Beijing Institute of Genomics, China)
- Chao Xie (National University of Singapore, Singapore)
- Hua Xu (Vanderbilt University, USA)
- Yu Xue (University of Science and Technology of China, China)
- Wenying Yan (Soochow University, China)
- Ueng-Cheng Yang (National Yang Ming University, Taiwan)
- Yan Zhang (CAS Shanghai Institutes for Biological Sciences, China)

- Guang Lan Zhang (Dana-Farber Cancer Institute, USA)
- Xing-Ming Zhao Tongji University, China)
- Zhongming Zhao (Vanderbilt University, USA)
- Xingming Zhao (Tongji University, China)
- Dongxiao Zhu (Wayne State University, USA)
- Shanfeng Zhu (Fudan University, USA)

Sub-reviewers:

- Sadnan Almanir (University of New Brunswick, Canada)
- Stefan Arold (King Abdullah University of Science and Technology, Saudi Arabia)
- Feixing Cheng (Vanderbilt University, USA)
- Rui Camacho (University of Porto, Portugal)
- Derin Keskin (Dana-Farber Cancer Institute, USA)
- Guoliang Li (The Jackson Laboratory, USA)
- Qian Liu (University of Technology Sydney, Australia)
- Zexian Liu (University of Science and Technology of China, China)
- Songjian Lusong (University of Pittsburgh, USA)
- Qin Ma (University of Georgia, USA)
- Mikhail Okun (University of Vienna, Austria)
- Alfredo Pulvirenti (University of Catania, Italy)
- Heiko Schmidt (University of Vienna, Austria)
- Mario Veloso (Anestesia, Reanimação e Neurologia, Lda., Portugal)
- Xiao Wang (Tongji University, China)
- Jiayan Wu (Bejing Institute of Genomics, China)
- Min Wu (Institute for Infocomm Research, A*STAR, Singapore)
- Hui Yu (Vanderbilt University, USA)
- Xing-Ming Zhao (Tongji University, China)
- Changbo Zhao (The Methodist Hospital Research Institute, USA)
